# Non-canonicaly recruited TCRαβCD8αα IELs recognize microbial antigens

**DOI:** 10.1038/s41598-018-29073-7

**Published:** 2018-07-18

**Authors:** Lukasz Wojciech, Edyta Szurek, Michal Kuczma, Anna Cebula, Wessam R. Elhefnawy, Maciej Pietrzak, Grzegorz Rempala, Leszek Ignatowicz

**Affiliations:** 1 Institute for Biomedical Sciences, Georgia State University, Atlanta, GA USA; 2 National University of Singapore, Department of Microbiology, Singapore, Singapore; 3 Department of Computer Science, Old Dominion University, Norfolk, VA 23529 USA; 4 Mathematical Biosciences Institute, Ohio State University, Columbus, OH USA

## Abstract

In the gut, various subsets of intraepithelial T cells (IELs) respond to self or non-self-antigens derived from the body, diet, commensal and pathogenic microbiota. Dominant subset of IELs in the small intestine are TCRαβCD8αα^+^ cells, which are derived from immature thymocytes that express self-reactive TCRs. Although most of TCRαβCD8αα^+^ IELs are thymus-derived, their repertoire adapts to microbial flora. Here, using high throughput TCR sequencing we examined how clonal diversity of TCRαβCD8αα^+^ IELs changes upon exposure to commensal-derived antigens. We found that fraction of CD8αα^+^ IELs and CD4^+^ T cells express identical αβTCRs and this overlap raised parallel to a surge in the diversity of microbial flora. We also found that an opportunistic pathogen (*Staphylococcus aureus*) isolated from mouse small intestine specifically activated CD8αα^+^ IELs and CD4^+^ derived T cell hybridomas suggesting that some of TCRαβCD8αα^+^ clones with microbial specificities have extrathymic origin. We also report that CD8ααCD4^+^ IELs and Foxp3CD4^+^ T cells from the small intestine shared many αβTCRs, regardless whether the later subset was isolated from Foxp3^CNS1^ sufficient or Foxp3^CNS1^ deficient mice that lacks peripherally-derived Tregs. Overall, our results imply that repertoire of TCRαβCD8αα^+^ in small intestine expends *in situ* in response to changes in microbial flora.

## Introduction

The intraepithelial layer of a small intestine is inhabited by several subsets of TCRαβ^+^ IELs with different expression profiles of CD4 and CD8 co-receptors, mostly unknown antigen specificities, function, and diverse origin^1^. Most TCRαβ^+^ IELs express CD8αα homodimers and can bind to classical MHC class I and epithelial cell-associated non-classical MHC molecules, including mouse thymic leukemia antigen (TL)^1,2^. It is anticipated that majority of TCRαβCD8αα^+^ IELs are thymus-derived, and this subset contribute to induction of tolerance to self-antigens, whereas peripherally-induced TCRαβCD8αα^+^ IELs co-express CD4 coreceptor and tend to recognize microbe-derived antigen(s). This notion is supported by the evidence that immature thymocytes from T cell receptor (TCR) transgenic mice differentiate to TCRαβ^+^CD8αα^+^ IELs when the former cells become exposed to a high dose of their cognate antigen^3,4^. In contrast, TCRαβCD8ααCD4^+^ IELs differentiation occurs upon contact with exogenous antigens instead of self-antigens, and therefore this subset is considered as an antigen-specific “adaptive” counterparts of innate-like TCRαβCD8αα^+^ IELs of thymic origin^5^. This paradigm is also corroborated by experiments which showed that germ-free (GF) mice and mice fed with an elementary diet lacking protein antigens had fewer intestinal TCRαβCD8ααCD4^+^ but normal number of TCRαβCD8αα^+^ IELs, suggesting that only former subset sustainability depends on microbiota or external antigens^6,7^. Notably, in GF mice the repertoire of TCRαβCD8αα^+^ IELs is surprisingly diverse, but it changes following microbial colonization, indicating that this IEL subset also evolves locally upon contact with antigens derived from commensal flora^8,9^. How cross talk between gut commensal flora and TCRαβCD8αα^+^ IELs is orchestrated is an outstanding question that must be answered to understand the relationship between dysbiosis and intestinal inflammation, and to design new therapeutic approaches^10^.

Not surprisingly, multiple experimental evidence suggests that specificity of αβTCRs for self or microbial antigens influences these cells lineage commitment^11^. First, cloning and retroviral expression of several αβTCRs from various CD8αα^+^ IEL clones showed that these receptors are unique, recognize various classical and non-classical MHC molecules for their selection and antigen recognition, and serve a nonredundant function in the gut^12^. This motion was further reinforced by results of cloning and re-expression of “unconventional” αβTCRs from CD4^−^CD8^−^ (DN) thymocytes that biased these cells differentiation to CD8αα^+^ IEL lineage, demonstrating that these cells development is guided by αβTCRs specificity^13^. Unexpectedly, thymocytes expressing monoclonal TCRαβ cloned from pTreg cells also differentiated to TCRαβCD8αα^+^ IELs lineage unless their precursor frequency in the thymus was low, which redirected their commitment to pTregs^14^. This result implied that Tregs and IELs can express common αβTCRs that can support commitment to both lineages depending on precursor frequency. Finally, pTregs have been found to be a main reservoir of induced TCRαβCD8ααCD4^+^ (iIELs) in the small intestine^7^, inferring that although differentiation of thymocytes or peripheral T cells to IELs is guided by their receptors specificity, other cues including clonal competition and cytokines also contribute to IEL’s precursors commitment.

Reportedly repertoires of conventional T cells and intraepithelial TCRαβCD8αα^+^ IELs isolated from the small intestine from individual mice can share identical αβTCRs, indicating that the two mucosal compartments may exchange individual T cell clones^9,15^. Although it is likely that an induction of CD8αα homodimer can be controlled by the αβTCR signal strength and the stronger the signal, the higher the level of CD8αα induction on primary effector cells, the specific ligands that induce this coreceptor locally in the gut remain unknown. Furthermore, because in appropriate environment anergic T cells become pTregs which then may convert to TCRαβCD8ααCD4^+^ IELs, it is possible that the repertoire of the latter subset is continuously amended in the periphery in response to encountered microbiota, their metabolites or food-derived antigens^7^. An identification of gut-specific ligands that are recognized by CD8αα^+^ IELs should help us understand how these cells become activated and in what circumstances they get involved in intestinal homeostasis. In the past, an origin of TCRαβCD8αα^+^ IELs has been investigated at the population rather than clonal level, and when individual IELs were examined these cells expressed identical, transgenic TCRαβ, whereas an all-inclusive fate mapping at the level of individual CD8αα clones that comprise this subset has not been performed.

Here, we used mice where T cells co-express a heterogenous but restricted and fully controllable αβTCR repertoire (TCR^mini^ mice) in addition to Foxp3^GFP^ reporter, to examine how contact with microbiota-derived antigens induces CD4^+^ T cells reprogramming to IELs *in vivo*. We report that: 1/Direct comparison of αβTCRs expressed on DN thymocytes and TCRαβCD8αα^+^ IELs showed that no more than 50% of IELs expressed TCRs shared with their anticipated thymic precursors 2/Upon an introduction of new commensal(s) strains to GF TCR^mini^ mice, both natural and induced TCRαβCD8αα^+^ IELs subsets responded by selective clonal expansions, 3/Introduction of a broad microbial flora resulted in increased similarity between intestinal CD4^+^ and both TCRαβCD8αα^+^ IELs subsets, which only in part depended on the presence of pTregs. 4/A microbial strain isolated from small intestine of TCR^mini^ mice and identified later as *Staphylococcus aureus*, activated both TCRαβCD4^+^ and TCRαβCD8αα^+^ derived T cell hybridomas, suggesting that some of TCRαβ^+^CD8αα^+^ IELs have peripheral origin and recognize microbial antigens. 5/Mice lacking pTregs had reduced diversity of TCRαβ CD8ααCD4^+^ but not TCRαβCD8αα^+^ IELs. Thus, our results suggest that outside of the main, intrathymic pathway of TCRαβCD8αα^+^ IELs differentiation, mature CD4^+^ T cells can acquire CD8αα expression and downregulate the original co-receptor.

## Results

**Figure 1:**
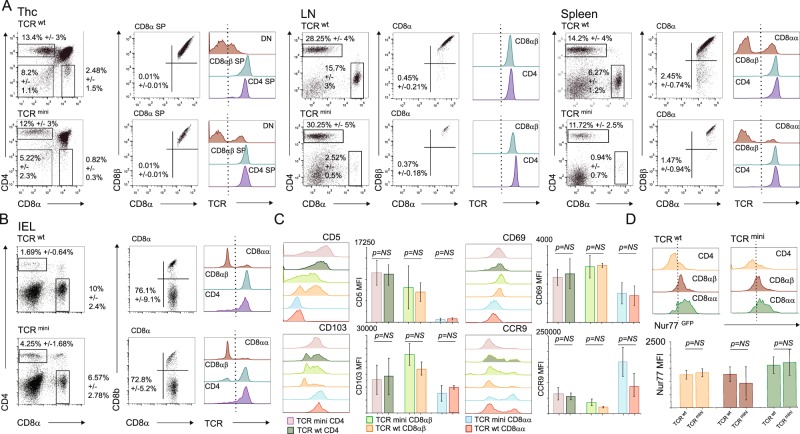
TCR^mini^ mice have normal development and homing of different subsets of αβTCR IELs. (**A**) Proportions and level of αβTCRs on thymocytes, CD4 and CD8 T cells in lymphatic organs of TCR^mini^ and WT B6 mice. (**B**) Proportions of T cells and IELs in the small intestine in TCR^mini^ and WT B6 mice. (**C**) Expression of selected activation and gut homing molecules on various subsets of IELs from TCR^mini^ and WT B6 mice. (**D**) Expression of Nur77^GFP^ reporter in IELs from TCR^mini^ or WT B6 mice.

Clonal diversity of αβTCRs on IELs in the small intestine is shaped by unknown self, diet and microbial antigens presented in the context of classical and non-classical MHC molecules^16^. To learn more about an origin of TCRαβCD8αα^+^ IELs and how microbial antigens impact these cells fate, we studied mice where T cells express heterogeneous but restricted TCR repertoire, (TCR^mini^) and Nur77^GFP^ labels antigen-triggered lymphocytes^17,18^. In TCR^mini^ mice timely expression of αβTCR guides natural commitment of thymocytes to various T effector subsets including CD4^+^Foxp3^+^ regulatory^17^ and IEL lineages. Accordingly, as shown in Fig. 1A approximately 1/3 of DN thymocytes in both TCR^mini^ and wild type mice expressed αβTCRs, albeit at slightly lower level as compared to CD4^+^ and CD8^+^ thymocytes presumably because these TCRs have higher affinities for self MHC/peptide complexes^19,20^. In the lymph nodes and spleen, most cytotoxic T cells represented conventional CD8αβ^+^ lineage, although in both strains a small subset of the TCRαβCD8αα^+^ IELs was spotted in their spleens (Fig. 1A). In contrast, in both strains TCRαβCD8αα^+^ cells constituted a predominant IELs population in the small intestine, where they accounted for over 70% of CD8^+^, and a remaining portion was split between CD4^+^ and CD4^+^CD8αβ^+^ subsets (Fig. 1B). All respective IEL subsets from TCR^mini^ and WT strains expressed comparable levels of TCRαβ, CD5 and CD69 as well as CD103 or CCR9 gut homing molecules, suggesting that expression of TCR^mini^ repertoire supports natural differentiation of various IEL lineages (Fig. 1B,C). Nur77^GFP^ reporter had its highest expression by TCRαβCD8αα^+^ IELs, which is compatible with a motion that these cells express αβTCRs with higher functional avidity (Fig. 1D)^4^. Overall, these observations suggested that in TCR^mini^ mice TCRαβCD8αα^+^ cells differentiate and home to the small intestine in a physiological manner, but how often these cells αβTCRs recognize intestinal antigens remain undetermined.

### Role of the small intestine microbiota in the reshaping of TCRαβCD8αα^+^ IELs repertoires

**Figure 2:**
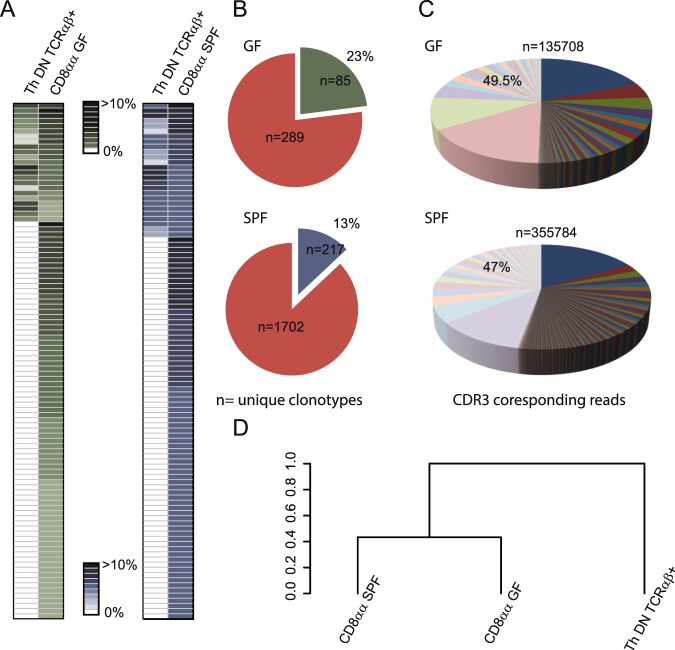
αβTCRs shared by DN αβTCR thymocytes and TCRαβCD8αα^+^ IELs account for approximately half of the TCRs expressed by these IEL subset in GF and SPF reared mice. (**A**) Distribution of 100 dominant αβTCRs expressed by CD8αα^+^ IELs in GF or SPF TCR^mini^ mice and their frequency on DNTCRαβ^+^ thymocytes. See SFig. 1 for the list of CDR3 sequences. (**B**) Contribution of αβTCRs shared by DN thymocytes and CD8αα^+^ IELs to the latter subset whole repertoire. (**C**) Relative proportion of TCRα sequences found on DN thymocytes and IELs in GF (upper pie chart) and SPF (lower pie chart) mice (n – total number of CDR3 reads). Separate piece of the pie is assigned to unique CDR3 sequence, and the size of the slice shows contribution to whole repertoire of CD8αα population in this strain of mice. Light colors are assigned to sequences shared with population of DN thymocytes. (**D**) Statistical analysis of similarity between αβTCRs expressed by IELs housed under GF and SPF conditions compared to DN thymocytes in SPF TCR^mini^ mice.

Reportedly, gross of TCRαβCD8αα^+^ IELs in the small intestine originate from immature thymocytes that downregulated both coreceptors upon selection by self-agonist/MHC peptide complexes^20^. Yet, it is not clear how clonal diversity of this repertoire is influenced by contact with food and enteric bacteria derived antigens, and whether this subset can expand by a recruitment of peripheral T cells. To examine a thymus imprint on the repertoire of TCRαβCD8αα^+^ IELs, we examined the repertoire of TCRα CDR3 regions expressed by these cells in GF and SPF mice, and then cross referenced these CDR3 sequences to the respective repertoire of TCRα CDR3 regions sequenced from sorted DN TCRαβ thymocytes. As shown in Fig. 2A, of 100 dominant αβTCRs expressed by TCRαβCD8αα^+^ IELs in GF mice 23 were also found within DN TCRαβ^+^ thymocytes compartment, and in SPF mice this number was similar (26 αβTCRs). When entire repertoires were compared, the αβTCRs shared by DN TCRαβ^+^ thymocytes and intestinal TCRαβCD8αα^+^ IELs constituted 23% in GF mice and 13% in SPF mice, and in both strains these cells accounted for approximately 50% of all sequences retrieved from TCRαβCD8αα^+^ IELs (Fig. 2B,C). Statistical analysis of these repertoires showed that TCRαβCD8αα^+^ IELs from germ free or SPF housed animals resembled more each other than the repertoire expressed by DN TCRαβ^+^ thymocytes (Fig. 2D). Likely more αβTCRs expressed by CD8αα^+^ IELs can be matched to respective αβTCRs on TCRαβCD4^dull^CD8^dull^ thymocytes that were not examined here, but it is also conceivable that some of these IELs have been recruited extrathymically.

### Colonization with microbial flora expands TCR repertoire of TCRαβCD8αα^+^ IELs

**Figure 3:**
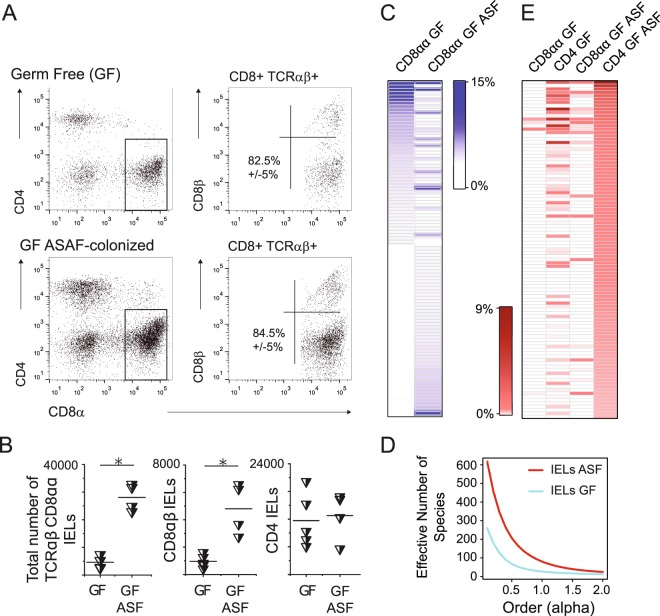
Colonization of GF TCR^mini^ mice with limited bacterial flora (ASF) increases the total number of TCRαβCD8αα^+^ IELs and recruits an additional IEL clones. (**A**) Proportions of various IEL subsets in GF TCR^mini^ and GF TCR^mini^ mice colonized with ASF. (**B**) Total number of IEL subsets in GF TCR^mini^ and GF TCR^mini^ mice colonized with ASF. (**C**) Distribution of dominant TCRs expressed by TCRαβCD8αα^+^ IELs in GF TCR^mini^ mice and GF TCR^mini^ mice colonized with ASF. (**D**) Diversity index of TCRαβCD8αα^+^ IELs from GF and SPF TCR^mini^ mice. (**E**) Allocation of dominant αβTCRs expressed in GF mice colonized with ASF on various subsets of IELs and T cells. For list of CDR3α sequences see SFig. 2.

Contact with commensal antigens can re-direct lineage commitment of mature CD4^+^ cells to IEL lineage, which results in formation of TCRαβCD8ααCD4^+^ IEL subset^5,7^. Thus, we examined how antigens derived from restricted commensal flora influence clonal diversity of TCRαβCD8αα^+^ IELs in GF TCR^mini^Foxp3^GFP^ mice colonized with Altered Schaedler Flora (ASF). This small consortium consists of eight, well defined microbial species and has been used to study plasticity in T cells lineage commitment upon an encounter of specific microbial flora^21^. As shown in Fig. 3A, B 6 weeks after colonization with ASF the proportion and total number of TCRαβCD8αα^+^ IELs increased significantly in colonized GF mice as compared to GF controls, and further analysis of αβTCR repertoire on IELs from these mice showed that introduced microbial antigens lead to an expansion of several clones expressing αβTCRs not found on a respective subset in GF TCR^mini^Foxp3^GFP^ mice. New TCRs not found on TCRαβCD8αα^+^ IELs in GF TCR^mini^ mice become abundant on this subset (Fig. 3C). Notably, many of these αβTCRs were also expressed by intestinal CD4^+^ T cells in both GF and colonized with ASF GF animals. In latter mice αβTCRs on CD4^+^ cells were more often shared with TCRαβCD8αα^+^ IELs suggesting that ASF presence was required for their recruitment to IELs subset. (Fig. 3C,E). Overall diversity of αβTCRsCD8αα^+^ IELs in GF mice colonized with ASF has also increased compared to respective subset from non-colonized mice, as depicted by higher values of diversity order (Fig. 3D). In sum, these results suggested that recognition of ASF-derived antigens by CD4^+^ T cells may recruit additional cells to TCRαβCD8αα^+^ IEL lineage.

**Figure 4:**
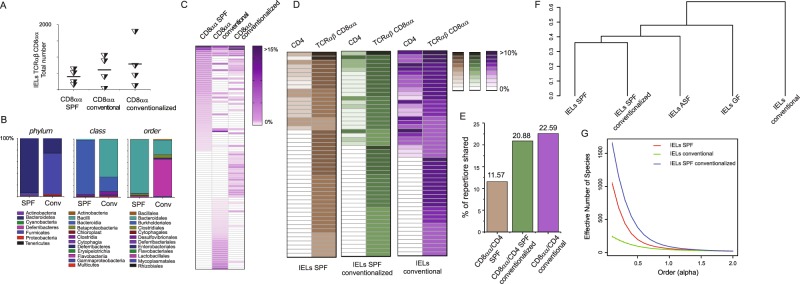
Conventionalization of SPF TCR^mini^ by co-housing or oral transfer of microbial flora increases an overlap between αβTCR repertoires expressed by intestinal CD4^+^ T cells and TCRαβCD8αα^+^ IELs. A/Total number of TCRαβCD8αα^+^ IELs in indicated strains of mice. (**B**) Distribution of major microbial constituents in the small intestine of SPF and conventionally reared TCR^mini^ mice based on 16S RNA analysis. (**C**) Conventionalization of TCR^mini^ mice by co-housing or oral transfer (infection) changes an allocation of dominant αβTCRs expressed by TCRαβCD8αα^+^ IELs. (**D**) In the small intestine of conventionalized TCR^mini^ mice CD4^+^ and TCRαβCD8αα^+^ IELs share more dominant αβTCRs than respective subsets in SPF TCR^mini^ mice. (**E**) Proportion of αβTCRs shared by CD8αα^+^ IELs and CD4^+^ T cells from the small intestine isolated from SPF, conventional and SPF conventionalized TCR^mini^ mice. (**F**) G/Statistical analysis of similarity and diversity indexes of αβTCRs expressed by IELs in TCR^mini^ mice inhabited with different microbial flora. TCRα CDR3 sequences depicted on heat maps in 4 C, D are listed in SFig. 3.

To further examine the role of microbe-derived antigens on IELs heterogeneity, we examined an impact of microbial flora on TCRαβCD8αα^+^ IELs in TCR^mini^ mice bred in conventional or barrier SPF facilities. We found that the total number of TCRαβCD8αα^+^ IELs recovered from TCR^mini^ mice bred in conventional rooms was only moderately higher as compared to the respective cell number from SPF TCR^mini^ mice (Fig. 4A). Conventionalization of the latter mice by 8 weeks co-housing also resulted in an increase in total number of TCRαβCD8αα^+^ IELs (Fig. 4A). A 16S rRNA sequencing of microbial flora from small intestine of these mice revealed that the microbiome from the small intestine of SPF TCR^mini^ mice predominated *Bacterioidales*, whereas *Lactobacillales* and *Clostridia* species were most common in the respective microbiome isolated from conventional TCR^mini^ mice (Fig. 4B). In addition, a routine screen for mouse opportunistic pathogens revealed presence of *Pseudomonas*, *Streptococcus* and *Helicobacter* species in conventional but not in SPF TCR^mini^ mice (Augusta University animal facility quarterly health reports). These observations suggested that some of commensals or opportunistic pathogens found in conventional mice support *in situ* expansion or differentiation of TCRαβCD8αα^+^ IELs.

To investigate whether non-overlapping species of microbiota from conventional mice alter αβTCRs allocation on CD8αα^+^ IELs, we used an oral gavage to transfer fresh content of small intestine from conventionally reared TCR^mini^ mice to SPF mice. Three weeks after this transfer the SPF TCR^mini^ mice colonized with flora from conventional TCR^mini^ animals were sacrificed, we isolated their IELs and examined their TCRα CDR3 repertoires. Though as previously noted the total number and proportions of IELs in mice reared in SPF or conventional facilities appeared similar (Fig. 4A), clearly the repertoires of TCRαβCD8αα^+^ IEL clones from TCR^mini^ mice reared in SPF vs in conventional facility appear dissimilar (Fig. 4C). In addition, many αβTCRs previously found only on IELs in conventional animals were now also expressed by TCRαβCD8αα^+^ cells in conventionalized SPF TCR^mini^ mice, which also increased an overlap between TCRα CDR3 repertoires on TCRαβCD8αα^+^ IELs and intestinal TCRαβCD4^+^ cells (Fig. 4D,E). Whereas in SPF TCR^mini^ mice approximately 12% of IELs and CD4^+^ cells from the small intestine expressed the same αβTCRs, this overlap almost doubled in TCR^mini^ mice inhabited with flora present in conventional facility (23%) (Fig. 4D,E). In addition, an analysis of the similarity indices allocated the repertoire of TCRαβCD8αα^+^ IELs from the short-term conventionalized SPF TCR^mini^ mice clustered with repertoires of TCRαβCD8αα^+^ IELs isolated from SPF, suggesting that introduced bacteria did not significantly changed this microbiome (Fig. 4F). We also noticed a substantial enrichment of the SPF-conventionalized repertoire with new clones (as reflected by a higher overall diversity of this repertoire (Fig. 4G)). These results suggested that intestinal bacteria or metabolites transferred from conventional mice to SPF-reared TCR^mini^ mice enhanced clonal diversity of TCRαβCD8αα^+^ IELs.

### *In vivo*, adoptively transferred TCRαβCD4^+^ T cells convert to TCRαβCD8αα^+^ IELs lineage upon interaction with agonist ligand bound to class II MHC

**Figure 5:**
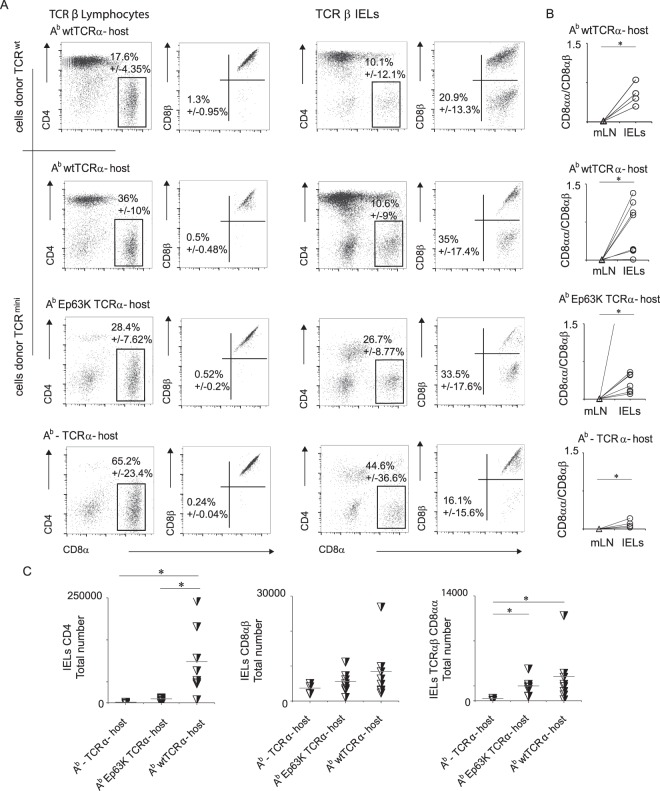
Some of transferred CD4^+^ T cells convert to TCRαβCD8αα^+^ IELs lineage. (**A**) Proportion of TCRαβCD8αα^+^ IELs recovered from the small intestine after cell transfer to different hosts. (**B**) TCRαβCD8αα^+^/TCRαβCD8αβ^+^ T cells ratio in the mesenteric LNs and small intestine epithelium after adoptive transfer of mature CD4^+^ T cells. Scale on Fig. 5B represents ratios of CD8αα/CD8αβ cells in host’s mesenteric lymph nodes (mLN) or small intestine epithelium, which have been derived from injected CD4^+^ lymphocytes. (**C**) Total numbers of TCRαβ CD4^+^, CD8αβ^+^ and CD8αα^+^ IELs recovered after cell transfer to indicated strains of mice.

Presented above results suggested that an expansion of repertoire expressed by TCRαβCD8αα^+^ IELs continues in the gut, and that this process involves recruitment of microbe-specific CD4^+^ cells. To further test this possibility, we sort-purified peripheral TCRαβCD4^+^ T cells from either wild type B6 or TCR^mini^Nur77^GFP^ mice (purity 98.6%) and adoptively transferred these cells into lymphopenic TCRα^−^ deficient, double deficient (TCRα^−^A^b−^) or TCRα^−^A^b^Ep63K recipients where all A^b^ molecules remain covalently bound with a single self-peptide (Ep63K). The Ep63K peptide is an analog of naturally occurring Eα(52–68) peptide with a single substitution in position 63, and the A^b^Ep63K complex is recognized as agonist by a fraction of CD4^+^ T cells derived from TCR^mini^ mice^22^. Only in the first type of recipient microbial antigens can bind to A^b^, whereas other recipients lacked A^b^ or have A^b^ bound with a single, covalently- linked self-peptide. Three weeks after transfer, we sacrificed all recipient mice and examined them for a presence of transferred CD4^+^ cells in small intestine and mesenteric lymph nodes (Fig. 5A). Interestingly, we found that clonal expansion of transferred CD4^+^ cells was well supported in the first two cohorts of TCRα^−^ recipients in their mesenteric LN and the small intestine epithelium, but only a small number of CD4^+^ cells survived in lymphopenic TCRα^−^A^b−^ recipients. These observations suggested that in the absence of class II MHC (TCRα^−^A^b−^ recipients) or upon contact with only a single self-peptide (TCRα^−^A^b^Ep recipients) most transferred CD4^+^ cells died off due to lack of subtle survival signals provided by their αβTCRs. In contrast, last two recipients had a sizable population of CD8αβ^+^ IELs residing in LNs and the epithelium of the small intestine (Fig. 5B,C), resembling reprogramming of CD4^+^ T cells to CD8αβ lineage in β2m-deficient mice inoculated with a tumor^23^. Notably, development of the CD8αα^+^ IELs was restricted to the small intestine epithelium (Fig. 5A,C) and abundance of this population was positively corelated with the expression of A^b^, regardless of the nature of A^b^-bound peptide(s).Therefore, reprogramming of CD4^+^ T cells to CD8αβ lineage most likely represents these cells alternative lineage commitment rather than a temporary phase that mirrors TCRαβCD8αα^+^ IELs phenotype^24^. Thus, these data support the hypothesis that peripheral recruitment of CD4^**+**^ T cells to TCRαβCD8αα^+^ IELs compartment depends on specific antigen(s) bound to class II MHC (A^b^).

### Identification of a specific microbial commensal(s) that recognition drive conversion of CD4^+^ IELs to TCRαβCD8αα lineage

**Figure 6:**
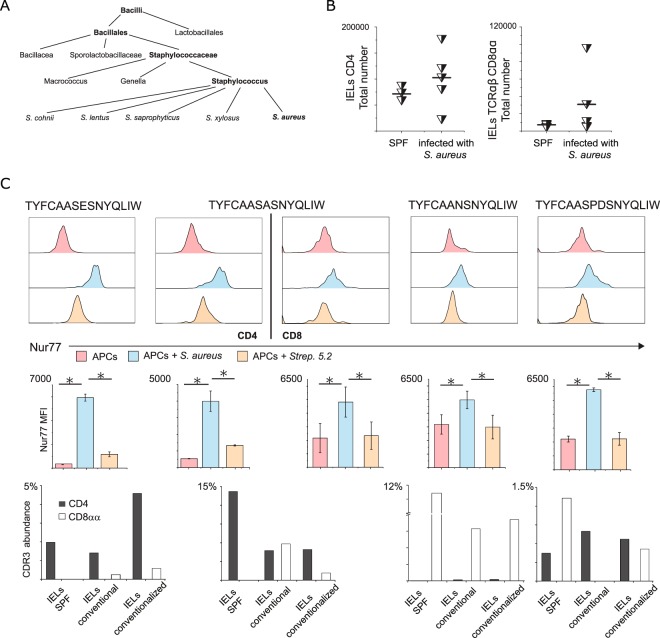
Inoculation of TCR^mini^ mouse with *S*. *aureus* drives conversion of CD4^+^ IELs to TCRαβCD8αα lineage. (**A**) Phylogenetic tree of *S*. *aureus* isolated from conventionally reared TCR^mini^ mice constructed based on comparison of 16S rRNA gene sequence and the concatenated sequences of publicly available *S*. *aureus* genome sequences downloaded from the NCBI including 48 completely assembled genomes. (**B**) Total numbers of CD4^+^ and TCRαβCD8αα^+^ IELs recovered after infection with *S*. *aureus*. (**C**) Selected TCRαβCD4^+^ and TCRαβCD8αα^+^ hybridomas respond to *S*. *aureus* lysate by upregulation of Nur77^GFP^ reporter expression. Overall, we tested 38 hybridomas derived from TCRαβCD8αα^+^ T cells. Abundance of CDR3 sequences from responding hybridomas in populations of TCR^mini^ mice with indicated microbiological status.

Since the highest proportion of CD4^+^ T cells and TCRαβCD8αα^+^ IELs sharing identical αβTCRs was found in conventionally reared TCR^mini^ mice, we hypothesized that microbe-derived antigens that bind to A^b^ and are exclusive for this colony facilitate conversion of intestinal CD4^+^ clones. To identify intestinal commensals that may provide these antigens, we propagated *ex vivo* several bacteria retrieved from conventionally reared TCR^mini^ mice. When these bacteria phylogenetic affiliation was revealed by 16S sequencing, we found that one sample represented an opportunistic pathogen *Staphylococcus aureus* (Fig. 6A) that was unique for conventionally but not SPF-reared TCR^mini^ mice. Next, we used this *S*. *aureus* isolate to infect SPF TCR^mini^ mice, but six weeks later an analysis of the small intestine epithelium showed no statistically significant changes in IELs proportion or these cells total number as compared to uncolonized control SPF mice (Fig. 6B). To test if IEL clones that may recognize *S*. *aureus* -derived antigens, we sorted TCRαβCD4^+^ and TCRαβCD8αα^+^ IELs from the small intestine of colonized SPF mice, then immortalized these cells by producing T cell hybridomas. Though CD8αα homodimer is not required for antigen recognition, to immortalize CD8αα^+^ IELs more efficiently we used BW thymoma that has been stably transduced with CD8α chain^25^. After two rounds of *ex vivo* activation with *S*. *aureus* isolate, we sorted polyclonal hybridomas with the highest expression of Nur77^GFP^ reporter, cloned them and established five (two TCRαβCD4^+^ and three TCRαβCD8αα^+^) hybridomas that showed elevated expression of Nur77^GFP^ reporter following co-culture with autologous APCs preincubated for 6 hours with *S*. *aureus* isolate, but not with control isolate from other bacteria (labelled as 5.2) (Fig. 6C). Notably, when we sequenced the CDR3 regions of the TCRα chains from *S*. *aureus* specific hybridomas, one of these αβTCRs was shared by both CD4^+^ and CD8αα^+^ specific hybridomas (Fig. 6C), supporting the view that CD4^+^ and TCRαβCD8αα^+^ IEL lineages can share the same antigen specificities. To further investigate this hypothesis, we cross referenced CDR3 sequences expressed by *S*. *aureus* -specific αβTCRs to the NGS database of αβTCRs expressed by IELs from SPF, SPF conventionalized and conventional TCR^mini^ mice. Although these CDR3 were represented in αβTCRs expressed by CD4^+^ T cells in all tested mice, the last two cohorts (conventional and SPF conventionalized) had same αβTCRs expressed by TCRαβCD8αα^+^ IELs (Fig. 6C), supporting the view these IELs have been reprogrammed to this lineage by contact(s) with specific microbial derivatives.

### Clonal diversity of TCRαβCD8αα^+^ and TCRαβCD8ααCD4^+^ IELs differentiating in mice lacking peripherally-derived CD4^+^Foxp3^+^ Tregs (pTregs)

**Figure 7:**
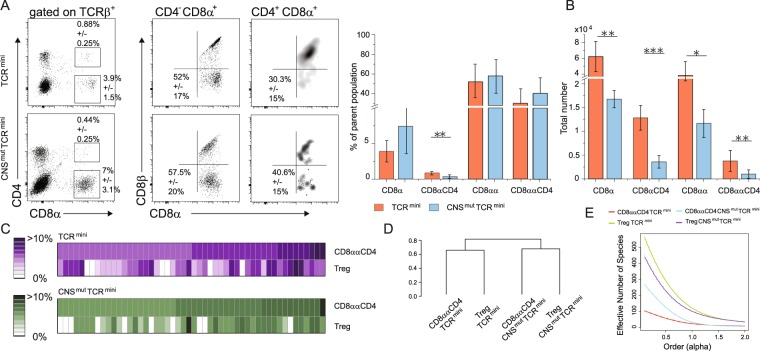
TCR^mini^CNS1^mut^ mice that lack pTregs have reduce number of TCRαβCD8αα^+^ IELs. (**A**) Proportions of TCRαβCD8αα^+^ and CD4CD8αα^+^ IELs in the small intestine of TCR^mini^ and TCR^mini^CNS1^mut^ mice. (**B**) Total number of TCRαβCD8^+^, CD4^+^CD8^+^, CD8αα^+^ and CD4^+^CD8αα^+^ IELs in indicated strains of mice. (**C**) Frequencies of dominant αβTCRs retrieved from TCRαβCD4CD8αα^+^ and TCRαβCD4Foxp3^GFP+^ from SPF TCR^mini^ and TCR^mini^ CNS1^mut^ mice. (**D**,**E**) Similarity and diversity indexes calculated for CD4Foxp3^+^Tregs and CD8ααCD4^+^ subsets isolated from TCR^mini^ and TCR^mini^CNS1^mut^ mice.

Reportedly, gross of CD8ααCD4^+^ IELs in the small intestine originate from CD4^+^Foxp3^+^ peripherally-derived regulatory cells (pTregs)^7^. Peripherally-derived Tregs (pTregs) are abundant in the colon, but sparse in the small intestine because upon entry to epithelial layer these cells downregulate Foxp3, retain CD4 and acquire CD8αα expression^26^. Therefore, we next examined whether mice that lack pTregs will continue to expand their CD8αα^+^ IELs repertoire via an extrathymic recruitment of TCRαβCD4^+^ cells. For this purpose, we crossed TCR^mini^Foxp3^GFP^ strain with pTregs-deficient CNS1^mut^ mice that harbor mutation in regulatory element controlling extrathymic induction of Foxp3^27^, and examined the number and clonal diversity of different subsets of IELs in the small intestine in these animals. As shown in Fig. 7A,B in agreement with previously published report^26^, TCR^mini^CNS1^mut^ mice had reduced total number of TCRαβCD8ααCD4^+^ cells in the small intestine as compared to respective subset in TCR^mini^CNS1^+^ control mice. However, the subset of TCRαβCD8αα^+^ IELs was also reduced although not as much as in case of TCRαβCD8ααCD4^+^ population, suggesting that pTregs may convert *in situ* to both TCRαβCD8αα^+^ subsets of IELs. Further comparison of dominant αβTCRs expressed by TCRαβCD4CD8αα^+^ IELs vs CD4^+^Foxp3^GFP+^ cells from small intestine of TCR^mini^ and TCR^mini^CNS^mut^ mice showed that in both strains many αβTCRs were shared by both lineages, irrespectively of the presence or absence of pTregs, although in the former strain the similarity between both repertoires appeared to be higher (Fig. 7C,D). An estimator of clonal diversity of αβTCRs expressed by different IEL and Treg subsets showed that whereas mucosal Tregs had broader repertoire in CNS1-sufficent TCR^mini^ mice, unexpectedly more diverse αβTCRs were expressed by CD8ααCD4^+^ IELs in TCR^mini^CNS^mut^ than by an equivalent subset from TCR^mini^ mice (Fig. 7E). In sum these results implied that though CD4Foxp3^+^ T cells my convert to IELs in the small intestine, the impact of this recruitment on the latter subset repertoire is likely limited.

## Discussion

Our study reveals yet another genus of Gram-positive bacterial strain (*S*. *aureus*) that in humans predominantly inhabits nose and skin, and in mice colonizes the small intestine^28^ and induces CD8αα expression on TCRαβCD4^+^cells. However, in contrast to other microbial species that have a similar ability to support T cells lineage conversion, a portion of *S*. *aureus* induced CD4^+^ clones lost this co-receptor expression. This observation is in odd with the current view that TCRαβCD8αα^+^ IELs exclusively originate from immature DN thymocytes rather than peripheral cells, and their antigen specificities encompass self rather than microbe-derived antigens^29–31^. In the past, IELs that specifically reacted to antigens derived from *Lactobacilli* or *Fecalibacterium* species were TCRαβCD8ααCD4^+^ DP IELs which upon contact with microbiota-derived antigens downregulated Thpok expression^32,33^. However, other reports showed that TCRαβCD8αα^+^ IELs can differentiate from peripheral mature T cells but the specific cues responsible for this route of conversion or whether αβTCRs recognition of commensals species was involved has not been addressed^34^.

We found no evidence that expression of the restricted αβTCR repertoire somewhat modified differentiation or phenotype of intestinal TCRαβCD8αα^+^ IELs in TCR^mini^ mouse model, as these cells expressed a comparable levels of homing receptors (CD103, α4β7), and activation markers (CD5, CD69 or Nur77^GFP^) as respective subsets isolated from wild type mice. These findings suggested that restriction of T cells clonal diversity implemented in this mouse model did not limit these cells ability to naturally differentiate to IEL subsets in the thymus or in the periphery. This conclusion is also supported by a detailed analysis of repertoires on αβTCR^+^ DN thymocytes and TCRαβCD8αα^+^ IELs. When these subsets were retrieved from GF mice they had 23% of dominant TCRs shared, which accounted for almost half of all αβTCRs sequences retrieved from TCRαβCD8αα^+^ IELs. Thus, thymic imprint on TCRαβCD8αα^+^ IELs subset was foremost visible, implicating their mutual origin. However, we also found TCRs exclusive for αβTCRCD8αα^+^ IELs, which were not expressed on their anticipated thymic precursors, and their proportion was even higher in mice that were housed under SPF conditions. An origin of IELs expressing those αβTCRs remained to be determined.

Unexpectedly the same αβTCRs were also sequenced from CD4^+^ cells, and their presence increased due to presence of microbes inhabiting TCR^mini^ in conventional facility. In this facility, the small intestine microbiome of TCR^mini^ showed an expansion of *Lactobacilli* at the cost of *Bacteroidetes*. Further analysis identified *S*. *aureus* as prominent, aerobic strain found only in conventional or conventionalized SPF but not SPF TCR^mini^ mice. Although this bacteria was originally described as natural inhabitant in the mouse intestine^35^, so far its impact on the stability of CD4^+^ T cells lineage commitment has not been examined. We found that bacterial isolates from *S*. *aureus* were recognized by T cell hybridomas derived from TCRαβCD4^+^ and TCRαβCD8αα^+^ IELs, of which several were retrieved from SPF TCR^mini^ mice co-housed with *Staphylococcus*-positive TCR^mini^ cohort. The co-housing TCR^mini^ SPF mice with their identical, conventional mice also increased the proportion of shared, abundant αβTCRs expressed by intestinal TCRαβCD8αα^+^ and TCRαβCD4^+^ IELs subsets.

The intestinal epithelium is unique in that it harbors auto-reactive T cells which αβTCRs are largely absent from the peripheral repertoire in normal mice, and physiological function of these T cells remains unclear. Similarly, to supplementation of daily diet with *L*. *reuteri* to combat inflammation via enhanced production of inosine and idole-3 lactic acid, discriminatory fermentation and growth patterns of selected, nonpathogenic *Streptococcus* strains can directly modulate mucosal immune responses by reprograming mature T cells to regulatory lineages. To this time point, elucidating the role of the small intestinal microbiota, especially of the abundant and diverse *Streptococcus* species and how their protein homologs and/or metabolites impact the immune response is a task for the future^36^.

## Materials and Methods

### Mice

The TCR^mini^ strains and mice with Foxp3^GFP^ reporter were produced as previously described^17^. In brief, The TCR^mini^ mice were obtained by crossing transgenic mice expressing Vα2 and Jα26 segments in germ line configuration with transgenic mice expressing TCRβ chain of the A^b^Ep63K-specific αβTCRs and TCRα- mice. The TCR^mini^ strain was also backcrossed with Nur77^GFP^ reporter mice obtained from The Jackson Laboratory. The TCRα-, A^b^- and C57BL/6 mice were purchased from Jackson Laboratory. Both females and males were used that were approximately 8 to 12 weeks old unless stated differently in the text. CNS1^mut^Foxp3^GFP^ (CNS1^mut^) strain was obtained by mating Foxp3^CNS1mut^ (received from Dr. Susan Schlenner (KU Leuven, Belgium)^37^) with C57BL/6Foxp3^GFP^ mice^38^. Only males were used from this strain. All animals were housed under specific pathogen-free conditions in the AU Animal Facility at Augusta, GA, and after we relocated our laboratory at GSU Animal Facility in Atlanta, GA. All experimental procedures were carried out in accordance with the relevant guidelines and regulations, which were reviewed and approved by the AU and GSU Institutional Animal Care and Use Committees.

### Phenotype analysis and cell sorting

Thymii, lymph nodes and spleens from individual mice were harvested and passed through 100 μm net to obtain single-cell suspension. To isolate IELs from small intestine, this organ was cut off, flushed with Ca^2+^Mg^2+^-free PBS to remove feces and mucus, which was followed by a removal of Peyer’s patches from the intestine wall. Next intestine was cut in pieces and shaken in Ca2^+^Mg2^+^-free HBSS which also contained 5% FBS, 2 mM EDTA, and 1 mM DTT for 15 min at 37°. Then IELs were further purified with Percoll gradient and the single-cell suspension was further analyzed by flow cytometry.

Cell surface staining with monoclonal antibodies was done by standard procedures. The following antibodies were used: anti-CD4 (clone GK1.5, 0.4 µg ml^−1^), anti-CD4 (clone RM4–5, 0.4 µg ml^−1^), anti-CD8α (clone 53–6.7, 0.4 µg ml^−1^), anti-CD8β (clone H35-17.2, 0.4 µg ml^−1^), anti-CD5 (clone 53-7.3, 0.4 µg ml^−1^), anti-CD69 (clone H1.2F3, 0.4 µg ml^−1^), anti-TCR Vα2 (clone B20.1, 0.33 µg ml^−1^), anti-TCR Vβ14 (clone 14-2, 1.25 µg ml^−1^), anti TCRαβ (clone H57-597, 0.5 µg ml^−1^). Intra-cellular staining was performed after treatment with cell fixation/permeabilization kit (eBioscience), according to the manufacturer’s protocol. Monoclonal antibodies were obtained from: BD Biosciences, BioLegend or eBioscience. After staining, cells were analyzed using FACSCanto (BD) or Cytoflex (Beckman) cytometer, and data were analyzed using Flowjo V10 (TreeStar Inc.).

For sorting, cells were stained with anti-CD4, anti-CD8α, anti-CD8β, anti-TCR Vα2. CD4^+^Foxp3^+^ and CD4^+^Foxp3^−^ subsets were separated based on expression of Foxp3^GFP^ reporter. All samples were sorted using either MoFlo cell sorter (Beckman Coulter) or Sony (SH800) sorter with purity above 98%.

### Synthesis of TCRα cDNA libraries and high throughput sequencing

Preparation of library for high throughput sequencing was performed as previously described^22^. Total RNA was isolated from the sorted subsets using RNeasey ^Mini^ Kit (Qiagen) according to the manufacturer’s protocol. Synthesis of the first complementary DNA (cDNA) strand was performed with a primer specific for the TCR Cα region (5′-TCGGCACATTGATTTGGGAGTC-3′) using Superscript III cDNA synthesis kit (Invitrogen). Incorporation of Ion Torrent sequencing primers (A-Kay and P1-Kay) to each TCR cDNA together with ‘bar-coding’ of DNA material was performed during the first amplification step using Accuprime Taq Polymerase (Invitrogen). The PCR reaction was carried out with a pair of primers specific to the Vα2 and Cα regions of the TCRα chain. The sequence of the 1st primer was as follows: 5′-**CATCCCTGCGTG TCTCCGACTCAG***XXXXXXXXXX*GACTCTCAGCCTGGAGACT-3′, where the embolden font highlights sequence specific to the Vα2 segment of TCR, italic font marks a bar-code sequence, and the bold font is a sequence of the forward sequencing primer A-Kay. The sequence of 2nd primer was as follows: 5′-**CCTCTCTATGGGCAGTCGGTGATTGGT**ACACAGCA GGTTCTG-3′, where embolden font highlights nucleotides specific to constant region of TCRα and bold font marks sequence of reverse sequencing primer P1-Kay. After denaturation (95 °C for 2 min) four cycles of PCR proceeded as follows: three cycles at 94 °C for 15 s/54 °C for 15 s/72 °C for 40 s and one cycle 94 °C for 15 s/58 °C for 15 s/72 °C for 40 s. The obtained cDNA was cleaned using AMPure XP kit (Beckman Coulter) and used for emulsion PCR amplification. PCR products were cleaned again with the AMPure XP kit and the molarity of each product containing various CDR3 libraries was calculated using quantitative PCR performed with A-Kay and P1-Kay primers, according to the manufacture’s protocol (Sybergreen PCR master mix, BioRad). The libraries were mixed in equal molarity, subjected to the final amplification in the same conditions as described above, and sequenced at EdgeBio Systems (Gaithersburg, MD, USA). Low quality reads and incomplete and erroneous sequences were eliminated during data processing using filters provided by Ion Torrent Suite software. All sequences were aligned to the constant Vα2 and Cα regions and examined as described.

### Adoptive transfer

We transferred intravenously 2 × 10^6^ of sorted CD4^+^CD25^−^Foxp3^GFP−^ cells from wild type or TCR^mini^ mice to different TCRα^−^ hosts. Mouse weight was monitored daily and the experiment was terminated when the recipient’s weight reached 80% of starting weight.

### T cell hybridomas

To identify antigen-specific αβTCRs, CD4^+^ or CD8αα IELs from TCR^mini^ mice were sort-purified and expanded *in vitro* for 3 (CD4^+^) or 7 (CD8) days in the presence of antiCD3 (10 µg ml^−1^) and IL-2 (50 U/ml). Production of the T cell hybridomas from CD4^+^ and CD8^+^ were previously described, with the modification that CD8αα^+^ IELs were fused with BW variant transfected with CD8αα as described^39,40^, and after fusion cultured in selecting medium that in addition to HAT contained 0.7 mg/ml of G418. Reponses of hybridomas to APCs presenting antigens derived from cecal or bacterial lysates were determined based on change in the relative expression of Nur77^GFP^ reporter using standard flow cytometry procedures, and by detecting the amount of IL-2 produced following activation. The two-sample t-test and one-way ANOVA test was used to calculate statistical significance.

### Statistical methods

Analysis was performed as previously described using the R statistical software (R 2.15.0)^22^. For the comparative analysis of the overlap a mutual information index (I-index) was used as defined^41^ together with a hierarchical (agglomerative) clustering procedure with Ward linkage method^42^. Stability of clustering was assessed via parametric (multinomial) bootstrap - the 95% confidence bounds for similarity matrices were constructed based on the Frobenius norm. The sample coverage (i.e. the conditional probability of discovering a new receptor – (CVG) was estimated according to the Good-Turing formula^41^. The CVG index was calculated using a resampling procedure (multinomial bootstrap) with varying sample sizes (from fifty up to the size of the original sample) to assess the effect of under-sampling on the analysis. Diversity was quantified using the effective number of species (ENS) and presented in the form of diversity profiles^41^. These profiles are plots of the order of the index versus the value of the index, thus enabling comparisons of diversity with more weight put on rare (the order of index lower than one) or abundant (the order of index higher than one) receptors. The 95% confidence bounds were constructed as described above. The significance of differences between individual samples or groups of mice was determined by two sample t- test and one-way ANOVA test using Origin 9.1 software, and 3 levels of statistical significance were used: p values < 0.1 < 0.05 and < 0.01.

### Isolation and characterization of *S*. *aureus* as an opportunistic pathogen in conventionally reared TCR^mini^ mice

Two conventional reared TCR^mini^ were sacrificed using carbon dioxide according to approved animal protocol, the content of their cecum was isolated and resuspended in phosphate-buffered saline in several concentrations. Next solutions were spread onto LB agar using sterile glass spatula, and plates were incubated under aerobic conditions (5% CO2) for 6 days at 37 °C. Culture purity of single colonies was assured by streaking twice onto agar and was examined by observing cell morphology after Gram-staining and colony morphology. DNA was extracted from washed bacterial cell pellets using the DNeasy for pretreatment of Gram-positive bacteria.

The 16S rRNA genes were amplified using appropriate primers^43^. Amplicons were purified using agarose gel electrophoresis and the Wizard SV Gel and PCR Clean-Up System (Promega) and sent to Genewiz US for sequencing. Sequences of closely related organisms were obtained using the BLAST function of the NCBI server. All sequences were aligned using the BioEdit software, version 7.0.5.3 Percentages of similarity were calculated after unambiguous alignment of each isolated sequence with those of the most closely related species, using the DNA Distance Matrix function of the BioEdit and SILVA rRNA database available at www.arb-silva.de.

All experimental protocols were approved by Augusta University or Georgia State University Biosaftety, Chemical and IACUC Institutional Committees. Methods were carried out in accordance with the relevant guidelines and regulations.

### Data Availability

All data generated or analysed during this study are included in this published article (and its Supplementary Information files).

All resources will be made available to the academic community consistent with the spirit of scientific collaboration. To request mice or reagents, please contact corresponding author. Genetically modified mouse strains will be made available to investigators at academic institutions conducting non-commercial research.

## Electronic supplementary material

Supplemental information
