# Supplementary material for: Non-canonicaly recruited TCRαβCD8αα IELs recognize microbial antigens
Source: Sci Rep. 2018 Jul 18;8:10848. doi: 10.1038/s41598-018-29073-7 (PMC6052027; doi:10.1038/s41598-018-29073-7)
Supplement: Supplementary file 1 — Supplemental information [file 41598_2018_29073_MOESM1_ESM.pdf]

Non-canonicaly recruited TCR$\alpha\beta$CD8$\alpha\alpha$ IELs recognize microbial antigens.

Lukasz Wojciech[2¶], Edyta Szurek[1¶], Michal Kuczma[1], Anna Cebula[1], Wessam R Elhefnawy[3], Maciej Pietrzak[4], Grzegorz Rempala[4], and Leszek Ignatowicz[1*].

1. **Institute for Biomedical Sciences, Georgia State University, Atlanta, GA, US**
2. **National University of Singapore, Department of Microbiology, Singapore.**
3. **Department of Computer Science, Old Dominion University, Norfolk, VA 23529, US**
4. **Mathematical Biosciences Institute, Ohio State University, Columbus, OH, US**

***To whom correspondence should be addressed.**
¶ These authors equally contributed to this work.

Supplemental figure legends:

SFig. 1 CDR3 sequences shown in Fig. 2A

SFig. 2 CDR3 sequences shown in Fig. 3C, E

SFig. 3 CDR3 sequences shown in Fig. 4C, D.

SFig. 4 Expression of αβTCR and CD8αα on microbe-specific hybridomas.

SFig. 5 CDR3 sequences shown in Fig. 7C

SFig. 1

GF	SPF
**TYFCAAHSNYQLIW**	**TYFCAANSNYQLIW**
**TYFCAGNTGGLS**	**TYFCAASPDSNYQLIW**
**TYFCAANSNYQLIW**	**TYFCAAHSNYQLIW**
**TYFCAARGNYQLIW**	**TYFCAASAASNYQLIW**
**TYFCAAKGSNYQLIW**	**TYFCAAGNYQLIW**
**TYFCAARGDTGGLS**	**TYFCADSNYQLIW**
**TYFCAARHSNYQLIW**	**TYFCAASASSNYQLIW**
**TYFCADSNYQLIW**	**TYFCAASAMDSNYQLIW**
**TYFCAASAGNYQLIW**	**TYFCAASEASNYQLIW**
**TYFCAASRSNYQLIW**	**TYFCAARGDTGGLS**
**TYFCAASAASNYQLIW**	**TYFCAAILNSNYQLIW**
**TYFCAATMDSNYQLIW**	**TYFCADMDSNYQLIW**
**TYFCAASPDSNYQLIW**	**TYFCAASSNYQLIW**
**TYFCAADSNYQLIW**	**TYFCAADSNYQLIW**
**TYFCAGMDSNYQLIW**	**TYFCAARDSNYQLIW**
**TYFCAATHSNYQLIW**	**TYFCAEDSNYQLIW**
**TYFCADMDSNYQLIW**	**TYFCAASSSNYQLIW**
**TYFCAASASSNYQLIW**	**TYFCAASGSNYQLIW**
**TYFCAAGNYQLIW**	**TYFCAASVDSNYQLIW**
**TYFCAASSNYQLIW**	**TYFCAVHSNYQLIW**
**TYFCAARDSNYQLIW**	**TYFCAASMDSNYQLIW**
**TYFCAASGSNYQLIW**	**TYFCAASACSNYQLIW**
**TYFCAASGEDSNYQLIW**	**TYFCAASGNTGGLS**
TYFCAASNYQLIW	**TYFCAASGMDSNYQLIW**
TYFCAASTYQLIW	**TYFCAAKDYQLIW**
TYFCAASDYQLIW	**TYFCAASWAGGLS**
TYFCAASAYQLIW	TYFCAASNYQLIW
TYFCAAMGSNYQLIW	TYFCAGSNYQLIW
TYFCAGSNYQLIW	TYFCAARSHSNYQLIW
TYFCAAYQLIW	TYFCAAINYQLIW
TYFCAASGYQLIW	TYFCVGSNYQLIW
TYFCAASEMCNYQLIW	TYFCAASDNSNYQLIW
TYFCAASDNYQLIW	TYFCAASHYQLIW
TYFCAVSNYQLIW	TYFCAATNYQLIW
TYFCAARMDTGGLS	TYFCAAMGSNYQLIW
TYFCAASGNYQLIW	TYFCAARRDTGGLS
TYFCAARYQLIW	TYFCAACNYQLIW
TYFCAAVNYQLIW	TYFCVGGNYQLIW
TYFCAASHYQLIW	TYFCAAENNYQLIW
TYFCAAINYQLIW	TYFCAGGNYQLIW
TYFCAANNYQLIW	TYFCAASSNSNYQLIW
TYFCAASPHYQLIW	TYFCAARNYQLIW
TYFCAALNYQLIW	TYFCAASGYQLIW
TYFCAARAKGNYQLIW	TYFCAASDNYQLIW
TYFCAARNYQLIW	TYFCAADNYQLIW
TYFCAAEGSNYQLIW	TYFCAASAYQLIW
TYFCAARGPYQLIW	TYFCAASDPSNYQLIW
TYFCAAIVYQLIW	TYFCAALNYQLIW
TYFCAASAGQLIW	TYFCALNYQLIW
TYFCAANPHSNYQLIW	TYFCAASTYQLIW
TYFCAASHNQLIW	TYFCAAHNYQLIW
TYFCAAHNYQLIW	TYFCAASEYQLIW
TYFCAVRYQLIW	TYFCAAYNYQLIW
TYFCAASDPSNYQLIW	TYFCAARHYQLIW
TYFCAAYHQLIW	TYFCAANNYQLIW
TYFCAASAGYQLIW	TYFCAVSNYQLIW
TYFCAARRDTGGLS	TYFCAAYQLIW
TYFCAARHYQLIW	TYFCAAFSNYQLIW
TYFCAAEKNYQLIW	TYFCARNYQLIW
TYFCAARYYQLIW	TYFCAARRKSNYQLIW
TYFCAARVDTGGLS	TYFCALNTGGLS
TYFCAASPHDYQLIW	TYFCAGYQLIW
TYFCAACNYQLIW	TYFCAASGYYQLIW
TYFCAAANYQLIW	TYFCAASGHYQLIW
TYFCAAEMDSNYQLIW	TYFCAANPHSNYQLIW
TYFCAASECSNYQLIW	TYFCAASANQLIW
TYFCAASNAGSNYQLIW	TYFCAAVHYQLIW
TYFCAKRWLDSNYQLIW	TYFCAASAHSNYQLIW
TYFCVASNYQLIW	TYFCAASDGRNYQLIW
TYFCAASASCNYQLIW	TYFCAAANYQLIW
TYFCAAESDSNYQLIW	TYFCAAVNYQLIW
TYFCAASNNYQLIW	TYFCAKNTGGLS
TYFCAATNYQLIW	TYFCAAKNYQLIW
TYFCAATDYQLIW	TYFCAAIGSNYQLIW
TYFCAASPYQLIW	TYFCAASARYQLIW
TYFCASSNYQLIW	TYFCAAWGYQLIW
TYFCAADLSNYQLIW	TYFCAASLYQLIW
TYFCAASDRSNYQLIW	TYFCAASTNTGGLS
TYFCAGKGDTGGLS	TYFCAASPGYQLIW
TYFCAANGNYQLIW	TYFCAASCYQLIW
TYFCVGSNYQLIW	TYFCAASAHQLIW
TYFCALSNYQLIW	TYFCAAIEGSNYQLIW
TYFCAARQGCNYQLIW	TYFCALSNYQLIW
TYFCAASDNSNYQLIW	TYFCAATGNYQLIW
TYFCAADNYQLIW	TYFCAAGYQLIW
TYFCAPSNYQLIW	TYFCAASNNYQLIW
TYFCAATGYQLIW	TYFCAASRCNYQLIW
TYFCAYSNYQLIW	TYFCAASDYQLIW
TYFCAASSYQLIW	TYFCAANGYQLIW
TYFCAGGNYQLIW	TYFCAACHYQLIW
TYFCASPVASNYQLIW	TYFCAAERSNYQLIW
TYFCAASSNSNYQLIW	TYFCAARLDQLIW
TYFCAASPGYQLIW	TYFCAYSNYQLIW
TYFCAAYNYQLIW	TYFCAASVSCNYQLIW
TYFCAASANYQLIW	TYFCAAMSRNYQLIW
TYFCAASTSSNYQLIW	TYFCAASANYQLIW
TYFCAAFSNYQLIW	TYFCAGNYQLIW
TYFCAAEGNYQLIW	TYFCAAFNYQLIW
TYFCAARLHSNYQLIW	TYFCAAKRATGGLS
TYFCAGNAGGLS	TYFCAGEDSNYQLIW

SFig2

A

	GF	GF-ASF
TYFCAARSHSNYQLIW	19.8	15.74
TYFCATGGSNYQLIW	16.36	0
TYFCAAVQIMDSNYQLIW	10.33	11.24
TYFCAASGSPSNNYQLIW	4.89	0.33
TYFCAAKRGNYQLIW	4.17	0.04
TYFCAAIMESNYQLIW	4.07	1.06
TYFCAAGGLS	2.8	0.21
TYFCAARMNIGGLS	2.33	0.45
TYFCAASRPTTQLIW	2.2	0.17
TYFCAAHGYSNQLIW	2.09	3.48
TYFCAACSYQLIW	1.76	0.08
TYFCAASMDGNYQLIW	1.72	0
TYFCAAGDTGGLS	1.63	0.32
TYFCATGRDTNQLIW	1.59	0
TYFCAANRDSNYQLIW	1.55	0.88
TYFCAAGFMDSNYQLIW	1.47	0
TYFCAAIPRNYQLIW	1.4	1.97
TYFCAAAIYSNYQLIW	1.34	0.03
TYFCAAHYCNYQLIW	1.16	2.05
TYFCAASRHYQLIW	1.09	0
TYFCAAKRSSYQLIW	1.07	0
TYFCAADRAANQLIW	0.89	0.14
TYFCAASMADSNYQLIW	0.86	0.1
TYFCAAAAYSNYQLIW	0.8	0.31
TYFCAAHGYSNYQLIW	0.75	0.43
TYFCAASARGNHQLIW	0.74	1.95
TYFCAASRSPSSNYQLIW	0.64	0.46
TYFCAANEEHYQLIW	0.62	0
TYFCAAVRSNYQLIW	0.6	1.36
TYFCTADSNYQLIW	0.53	1.77
TYFCAAGTGTGGLS	0.49	0.65
TYFCAAMGTYQLIW	0.43	0
TYFCAAMRTYQLIW	0.42	0
TYFCAARARSNYQLIW	0.39	0
TYFCADRWSNYQLIW	0.37	0
TYFCAAHNSNYQLIW	0.34	1.04
TYFCAARQHSNYQLIW	0.33	0
TYFCAASGEVDSNYQLIW	0.33	0.1
TYFCAAMGIASYQLIW	0.3	0
TYFCATHDSNYQLIW	0.29	0.25
TYFCAAMHSNYQLIW	0.29	0
TYFCAAKEYQLIW	0.28	0.21
TYFCAASRGCYSNYQLIW	0.27	2.47
TYFCAASRARRNYQLIW	0.26	0.42
TYFCAARWASNYQLIW	0.26	0.01
TYFCAANPHSNHQLIW	0.25	0
TYFCAAENNQLIW	0.21	0
TYFCATNSGGLS	0.16	0
TYFCAAMRHTQLIW	0.13	0.09
TYFCAARWGYQLIW	0.1	0
TYFCAASRIRNYQLIW	0.01	0.22
TYFCAASTNSNYQLIW	0	0.22
TYFCAASAQTGGLS	0	0.24
TYFCAAGTSSNYQLIW	0	0.24
TYFCAASQSNQLIW	0.1	0.24
TYFCAVTAYQLIW	0.01	0.25
TYFCAAHDHNQLIW	0	0.26
TYFCAAPRADMDSNYQLIW	0	0.28
TYFCAASAGDGNYQLIW	0	0.28
TYFCAARWHSNYQLIW	0.01	0.28
TYFCAACVGDSNYQLIW	0.01	0.29
TYFCAASTGTGGLS	0	0.29
TYFCAAIDEHYQLIW	0.01	0.29
TYFCAADLLRNYQLIW	0.02	0.31
TYFCAASTADSNYQLIW	0	0.31
TYFCAATPLFSNYQLIW	0.01	0.32
TYFCAASPNTGGLS	0	0.33
TYFCAASKQSNYQLIW	0	0.34
TYFYARERNYQLIW	0	0.34
TYFCAAGWSTNQLIW	0	0.35
TYFCAAGDDMGSNYQLIW	0.01	0.37
TYFYAASALVSNYQLIW	0	0.37
TYFCAAMVSNYQLIW	0	0.37
TYFYAATMDSNYQLIW	0	0.38
TYFCAAGSAHQLIW	0	0.38
TYFCAADTHQLIW	0.03	0.41
TYFCAAGTNSNYQLIW	0	0.44
TYFCAADDAHQLIW	0	0.46
TYFWNSNYQLIW	0	0.46
TYFCAAHYSDYQLIW	0	0.46
TYFCAAGDDNYQLIW	0	0.5
TYFCAASAPVNYQLIW	0	0.56
TYFCAASGRDSNYQLIW	0	0.63
TYFCASKDTGGLS	0	0.65
TYFCAADQHNQLIW	0	0.67
TYFCAAKEGNYQLIW	0	0.68
TYFCAAIMDSKYQLIW	0.01	0.69
TYFCAAEEHNQLIW	0	0.72
TYFCAAHEHHQLIW	0.1	0.98
TYFSAASASNYQLIW	0	1.01
TYFCAASNSYQLIW	0	1.01
TYFCAAMCNYQLIW	0	1.07
TYFCAAIHDTQYQLIW	0	1.16
TYFCAACSQGDSNYQLIW	0	1.19
TYFCAARKAYQLIW	0.01	1.2
TYFCAAWRSSNYQLIW	0	1.21
TYFCAAVGHYQLIW	0.05	1.21
TYFCAAWMSTNQLIW	0.03	1.24
TYFCAASQAYQLIW	0	1.39
TYFCAAIPMDSNYQLIW	0.09	1.74

B

	GF		GF-ASF	
	CD8αα	CD4	CD8αα	CD4
TYFCAASAPVNYQLIW	0	2.81	0.52	9.46
TYFCAARCRQYQLIW	0	0	0	5.48
TYFCATSAASNYQLIW	0	1.95	0	3.52
TYFCAAIMDSKYQLIW	0.01	0.77	0.64	2.96
TYFCAANKGNNYQLIW	0	0.89	0	2.63
TYFCAARDSNYQLIW	0	1.62	0	2.28
TYFCAAVYANYQLIW	0	0.01	0	2.06
TYFCAAEMDSNYQLIW	0.01	7.46	0.17	2.02
TYFCAAGDEHYQLIW	0	0.49	0.11	1.96
TYFCAASRARRNYQLIW	0.25	5.59	0.39	1.75
TYFCAAHGYTTQLIW	0.1	0.12	1.14	1.5
TYFCAAKVTYQLIW	0	5.6	0.1	1.48
TYFCAASMADSNYQLIW	0.82	1.45	0.09	1.11
TYFCAASAGDGNYQLIW	0	0.35	0.26	1.09
TYFCAADGTYQLIW	0	0	0	1.01
TYFCASPRIGNYQLIW	0	0	0	0.98
TYFCASKSKHYQLIW	0	5.21	0.15	0.93
TYFCAAGIHYQLIW	0	0.06	0	0.92
TYFCAASRNYQLIW	0	0.11	0	0.84
TYFCAARVPCNYQLIW	0	0.17	0	0.81
TYFCASNTGGLS	0	0.04	0	0.8
TYFCATDSNYQLIW	0	0	0	0.74
TYFCAANPGSNYQLIW	0	0.37	0.02	0.69
TYFCADAHYQLIW	0	0.13	0	0.64
TYFCAANPHSNCQLIW	0	0.08	0	0.63
TYFCAADSTNQLIW	0	0	0	0.63
TYFCAASKNSNHQLIW	0	0.08	0	0.61
TYFCAAIPPIGGLS	0	0	0	0.61
TYFCADLRTGGLS	0.01	0.12	0	0.59
TYFCASKDGHYQLIW	0	0.15	0	0.55
TYFCAASPWDNYQLIW	0	0.84	0	0.54
TYFCAASASSNYQLIW	0	0.08	0.27	0.54
TYFCASKDGNYQLIW	0	0.01	0	0.53
TYFCAAWSPTYQLIW	0	0.51	0	0.52
TYFCGGRSNYQLIW	0	0.1	0	0.52
TYFCAARSWGMDSNYQLIW	0	0.13	0	0.52
TYFCGRADSNYQLIW	0	0	0	0.51
TYFCAADQHNQLIW	0	0.8	0.62	0.51
TYFCASKSNYQLIW	0	0	0	0.45
TYFCASKDSNYQLIW	0	0.03	0	0.41
TYFCAAREEQHYQLIW	0	0	0	0.39
TYFCAASHRRFNYQLIW	0	0	0	0.39
TYFCAVSAGTGGLS	0	0	0	0.39
TYFCAAVYIQLIW	0	0.28	0	0.39
TYFCAATVTYQLIW	0	0	0	0.37
TYFCAANSHYQLIW	0	0	0	0.37
TYFCAGTQRNQLIW	0	0	0	0.37
TYFCAEKRNYQLIW	0	0.07	0	0.35
TYFCAARIGGSNYQLIW	0	0.09	0	0.34
TYFCADRGNYQLIW	0	0	0	0.33
TYFYAATMDSNYQLIW	0	0.03	0.36	0.33
TYFCAARSWSSNYQLIW	0	0.32	0	0.33
TYFCRAVHYQLIW	0	0.38	0	0.32
TYFCQPDSNYQLIW	0	0	0	0.32
TYFCAVVSNYQLIW	0	0	0	0.32
TYFCVASASSNYQLIW	0	0	0	0.31
TYFCAAWDDSNYQLIW	0	0	0	0.31
TYFCGRHMSNYQLIW	0	0	0	0.3
TYFCAASAITTYQLIW	0	0	0	0.3
TYFCAASVDRINYQLIW	0	0	0	0.29
TYFCAASRDRSNYQLIW	0	0	0	0.29
TYFCAARVGSNYQLIW	0	0.2	0	0.29
TYFCAASVIGNYQLIW	0	0	0	0.29
TYFCAAEGDSNHQLIW	0	0.42	0	0.28
TYFCAADANQLIW	0.01	0.06	0	0.28
TYFCAARPSNYQLIW	0	0	0	0.28
TYFCAPSSYQLIW	0	0	0	0.27
TYFCAADHYQLIW	0	0.19	0	0.27
TYFCAASFEFWVSNYQLIW	0	0	0	0.27
TYFCAASEGGNYQLIW	0	0	0	0.26
TYFCGRRYNYQLIW	0	0.05	0	0.26
TYFCAADAYQLIW	0	0	0	0.26
TYFCAADKSNQLIW	0	0	0	0.26
TYFCAANPDSTNQLIW	0	0.04	0	0.26
TYFCAAQCNYQLIW	0	0	0	0.26
TYFCADDDSNYQLIW	0	0	0	0.25
TYFCAADTSSQLIW	0	0.13	0.08	0.25
TYFCASIDDAHQLIW	0	0	0	0.25
TYFCGSKPMDSNYQLIW	0	0	0	0.24
TYFCGSRSATNQLIW	0	0	0	0.24
TYFCAAGDDMGSNYQLIW	0.01	0.15	0.34	0.23
TYFCARVDTNQLIW	0	0	0	0.23
TYFCGSRYNYQLIW	0	0.02	0	0.23
TYFCAADQSGGLS	0	0.01	0.05	0.23
TYFCAASFGGLS	0	0.18	0	0.23
TYFCARETHYQLIW	0	0	0	0.22
TYFCAASDSDYQLIW	0	0.01	0.05	0.22
TYFCAADANTQLIW	0	0.25	0	0.22
TYFCAASPHIQLIW	0	0	0	0.22
TYFRAARSNYQLIW	0	0.05	0	0.21
TYFCAAVGHYQLIW	0.05	0.01	1.13	0.21
TYFCAADARNYQLIW	0	0.03	0	0.21
TYFCAASFGGLS	0	0	0	0.21
TYFCAATTYQLIW	0	0.02	0	0.21
TYFCARERATNQLIW	0	0.14	0	0.2
TYFCAASRGYCNYQLIW	0	0	0	0.2
TYFCAASATGGLS	0.01	0.14	0	0.2
TYFCAASPDGNYQLIW	0	0	0	0.2
TYFCAARGWHYQLIW	0	0.01	0	0.2
TYFCAASQAYQLIW	0	0	1.28	0.2

SFig3

A

	SPF	SPF conventionalized	SPF conventional
TYFCAASNYQLIW	16.813	9.852	14.64
TYFCAANSNYQLIW	11.461	3.162	17.712
TYFCAASAASNYQLIW	5	0.686	1.301
TYFCAAGNYQLIW	2.813	2.543	1.861
TYFCAGSNYQLIW	2.547	2.455	4.668
TYFCAARSHSNYQLIW	2.273	0	0
TYFCAAHSNYQLIW	2.079	0	1.286
TYFCAAINYQLIW	2.021	0	0.825
TYFCADSNYQLIW	1.98	5.871	0.515
TYFCVGSNYQLIW	1.766	0	0.855
TYFCAASDNSNYQLIW	1.71	0	0.9
TYFCAASHYQLIW	1.705	4.943	1.831
TYFCAATNYQLIW	1.496	0	0.515
TYFCAAMGSNYQLIW	1.426	0	0.409
TYFCAARRDTGGLS	1.328	0	0
TYFCAACNYQLIW	1.255	1.758	0.757
TYFCVGGNYQLIW	1.233	0	0
TYFCAASPDSNYQLIW	1.232	0	0.893
TYFCAAENNYQLIW	1.129	0	0
TYFCAGGNYQLIW	1.008	0	0
TYFCAASASSNYQLIW	0.997	0	1.778
TYFCAASSNSNYQLIW	0.976	0	0
TYFCAARNYQLIW	0.937	0.94	0.507
TYFCAARGDTGGLS	0.884	0.254	0
TYFCAASGYQLIW	0.812	1.471	0.575
TYFCAASDNYQLIW	0.81	0	0.878
TYFCAADNYQLIW	0.8	0.597	0
TYFCAASAYQLIW	0.783	0	0.794
TYFCAASAMDSNYQLIW	0.73	0	0
TYFCAASDPSNYQLIW	0.709	0	0
TYFCAALNYQLIW	0.707	0	0.59
TYFCALNYQLIW	0.704	0	0
TYFCAASEASNYQLIW	0.691	1.836	0
TYFCAASTYQLIW	0.686	0	0
TYFCAAHNYQLIW	0.666	0	0
TYFCAASEYQLIW	0.662	0	0
TYFCAAYNYQLIW	0.643	0	0
TYFCADMDSNYQLIW	0.574	0	0
TYFCAARHYQLIW	0.573	0	0
TYFCAANNYQLIW	0.546	2.344	0.726
TYFCAAILNSNYQLIW	0.531	10.847	0
TYFCAVSNYQLIW	0.485	0	1.158
TYFCAAYQLIW	0.483	0	0
TYFCAAFSNYQLIW	0.45	0	0
TYFCAEDSNYQLIW	0.429	0	0
TYFCARNYQLIW	0.428	0	0
TYFCAASSNYQLIW	0.415	0	0
TYFCAADSNYQLIW	0.411	0.288	1.173
TYFCAARRKSNYQLIW	0.404	0	0
TYFCALNTGGLS	0.379	0	0
TYFCAASATVSYQLIW	0	0	0.477
TYFCAAANYQLIW	0	0	1.226
TYFCAAFNYQLIW	0	0	0.431
TYFCAARGNQLIW	0	0	0.56
TYFCAASGEDSNYQLIW	0	0	0.484
TYFCAASGNQLIW	0	0	0.507
TYFCAASGNYQLIW	0	0	1.642
TYFCAASDYQLIW	0	0	3.624
TYFCAATDYQLIW	0	0	0.651
TYFCARDSNYQLIW	0	0	0.53
TYFCAAKGSNYQLIW	0	0	0.961
TYFCAARYYQLIW	0	0	0.666
TYFCAANHYQLIW	0	0	0.742
TYFCAGNTGGLS	0	0	1.801
TYFCAAEDSTGGLS	0	0	0.477
TYFCAGYQLIW	0	0	0.598
TYFCAASRSNYQLIW	0	0	1.143
TYFCALSNYQLIW	0	0	0.499
TYFCAASNAGSNYQLIW	0	0	1.665
TYFCASSNYQLIW	0	0	1.74
TYFCAASNNYQLIW	0	0	1.135
TYFCAARQGCNYQLIW	0	0	0.575
TYFCAASLNTGGLS	0	0.199	0
TYFCAASEKNYQLIW	0	0.221	0
TYFCAARDSNYQLIW	0	0.243	0.409
TYFCAGYSNYQLIW	0	0.243	0
TYFCAARGSNYQLIW	0	0.254	0
TYQLIW	0	0.31	0
TYFCAAMDSNYQLIW	0	0.332	0.885
TYFCAANRNSNYQLIW	0	0.332	0
TYFCAASKGSNYQLIW	0	0.343	0
TYFCAANYQLIW	0	0.354	0
TYFCAASESNYQLIW	0	0.387	0
TYFCAASACSNYQLIW	0	0.442	0
TYFCAASAVSNYQLIW	0	0.442	0
TYFCAASGSNYQLIW	0	0.464	0.439
TYFCAPGNYQLIW	0	0.487	0
TYFCEGDSNYQLIW	0	0.498	0
TYFCAASWGSSNYQLIW	0	0.52	0
TYFCAASTGGLS	0	0.564	0
TYFCAASPWDNYQLIW	0	0.63	0
TYFCAASTPCSNYQLIW	0	0.763	0
TYFCAAAGGLS	0	0.962	0
TYFCAASAKSNYQLIW	0	1.172	0
TYFCAASSYSNYQLIW	0	1.183	0
TYFCAASARDSNYQLIW	0	1.349	0
TYFCAASGNTGGLS	0	1.681	0
TYFCLGSNYQLIW	0	1.935	0
TYFCARLLGDSNYQLIW	0	2.035	0
TYFCAANPHSNYQLIW	0	2.057	0
TYFCAARCRRNYQLIW	0	2.223	0
TYFCAAGFYQLIW	0	2.245	0
TYFCAASPYQLIW	0	2.344	0.552
TYFCAASASNYQLIW	0	3.981	0
TYFCAASSNQLIW	0	4.467	0
TYFCAASVYQLIW	0	6.933	0

B

SPF	SPF conventionalized	SPF conventional
**TYFCAASNYQLIW**	**TYFCAASNYQLIW**	**TYFCAAILNSNYQLIW**
**TYFCAANSNYQLIW**	**TYFCAASDSNYQLIW**	**TYFCAASNYQLIW**
**TYFCAASPDSNYQLIW**	**TYFCAAGNYQLIW**	**TYFCAASVYQLIW**
**TYFCAASAASNYQLIW**	**TYFCAANSNYQLIW**	**TYFCAASASNYQLIW**
**TYFCAASHYQLIW**	**TYFCAGSNYQLIW**	**TYFCAASGNTGGLS**
**TYFCAARRDTGGLS**	**TYFCAAHSNYQLIW**	**TYFCAASAKSNYQLIW**
**TYFCAARSHSNYQLIW**	**TYFCAALNYQLIW**	**TYFCAASHYQLIW**
**TYFCAAHSNYQLIW**	**TYFCAASDYQLIW**	**TYFCAASEASNYQLIW**
**TYFCAASDNSNYQLIW**	**TYFCAATGYQLIW**	**TYFCARLLGDSNYQLIW**
**TYFCAASASSNYQLIW**	**TYFCAASAASNYQLIW**	**TYFCAANSNYQLIW**
**TYFCAASAMDSNYQLIW**	**TYFCAAINYQLIW**	**TYFCAANNYQLIW**
**TYFCAASSNSNYQLIW**	**TYFCAAPDSNYQLIW**	**TYFCAGSNYQLIW**
**TYFCAADNYQLIW**	**TYFCAASDNSNYQLIW**	**TYFCAASAASNYQLIW**
**TYFCAALNYQLIW**	**TYFCAASSNSNYQLIW**	**TYFCAADNYQLIW**
**TYFCAANNYQLIW**	**TYFCAAVNYQLIW**	**TYFCAAAGGLS**
**TYFCAAILNSNYQLIW**	**TYFCAASEASNYQLIW**	**TYFCAARNYQLIW**
**TYFCAEDSNYQLIW**	**TYFCAASGYQLIW**	**TYFCAAMDSNYQLIW**
**TYFCAADSNYQLIW**	**TYFCADSNYQLIW**	**TYFCAASESNYQLIW**
**TYFCAASSNYQLIW**	**TYFCAASTYQLIW**	**TYFCEGDSNYQLIW**
TYFCAAGNYQLIW	**TYFCAASPDSNYQLIW**	**TYFCAARDSNYQLIW**
TYFCAGSNYQLIW	**TYFCAAEGDSNYQLIW**	**TYFCAASAGNYQLIW**
TYFCAAINYQLIW	**TYFCAASASSNYQLIW**	**TYFCAGYSNYQLIW**
TYFCADSNYQLIW	**TYFCAASRSNYQLIW**	**TYFCAADSNYQLIW**
TYFCVGSNYQLIW	TYFCAAEPYSNYQLIW	**TYFCAASGSNYQLIW**
TYFCAATNYQLIW	TYFCAACNYQLIW	**TYFCAASAVSNYQLIW**
TYFCAAMGSNYQLIW	TYFCAADNYQLIW	**TYFCAARGSNYQLIW**
TYFCAACNYQLIW	TYFCAPSNYQLIW	TYFCADSNYQLIW
TYFCVGGNYQLIW	TYFCAAFNYQLIW	TYFCAAGNYQLIW
TYFCAAENNYQLIW	TYFCAASHYQLIW	TYFCAASPYQLIW
TYFCAGGNYQLIW	TYFCAANNYQLIW	TYFCAAGFYQLIW
TYFCAARNYQLIW	TYFCARNTGGLS	TYFCAARCRRNYQLIW
TYFCAARGDTGGLS	TYFCAASAYQLIW	TYFCAANPHSNYQLIW
TYFCAASGYQLIW	TYFCAATNYQLIW	TYFCLGSNYQLIW
TYFCAASDNYQLIW	TYFCAAENYQLIW	TYFCAACNYQLIW
TYFCAASAYQLIW	TYFCAAISDSNYQLIW	TYFCAASGYQLIW
TYFCAASDPSNYQLIW	TYFCAASLYQLIW	TYFCAASARDSNYQLIW
TYFCALNYQLIW	TYFCAAMGYQLIW	TYFCAASSYSNYQLIW
TYFCAASEASNYQLIW	TYFCAANPDSNYQLIW	TYFCAASSNQLIW
TYFCAASTYQLIW	TYFCAASANQLIW	TYFCAASTPCSNYQLIW
TYFCAAHNYQLIW	TYFCAASGCGGLS	TYFCAASPWDNYQLIW
TYFCAASEYQLIW	TYFCAASASNSNYQLIW	TYFCAASTGGLS
TYFCAAYNYQLIW	TYFCAARGEDSNYQLIW	TYFCAASWGSSNYQLIW
TYFCADMDSNYQLIW	TYFCGGSNYQLIW	TYFCAPGNYQLIW
TYFCAARHYQLIW	TYFCAASQLIW	TYFCAASACSNYQLIW
TYFCAVSNYQLIW	TYFCAATSASNYQLIW	TYFCAANYQLIW
TYFCAAYQLIW	TYFCAASYYQLIW	TYFCAASKGSNYQLIW
TYFCAAFSNYQLIW	TYFCAVSNYQLIW	TYFCAANRNSNYQLIW
TYFCARNYQLIW	TYFCAAETDSNYQLIW	TYQLIW
TYFCAARRKSNYQLIW	TYFCAARSASNYQLIW	TYFCAARGDTGGLS
TYFCALNTGGLS	TYFCAASDASNYQLIW	TYFCAASEKNYQLIW

SFig4

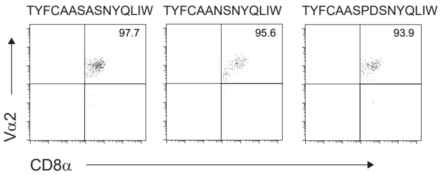
TYFCAASASNYQLIW
TYFCAANSNYQLIW
TYFCAASPDSNYQLIW
97.7
95.6
93.9
Vα2
CD8α

SFig5

TCR^{mini}	CNS^{mut} TCR^{mini}
TYFCAASPDTYQLIW	TYFCAASAYSNYQLIW
TYFCAASESNYQLIW	TYFCAASESNYQLIW
TYFCAASPLDSNYQLIW	TYFCAARDSNYQLIW
TYFCAASDSNYQLIW	TYFCAAVNTGGLS
TYFCAARDSNYQLIW	TYFCAASNTGGLS
TYFCARERNYQLIW	TYFCAASASNYQLIW
TYFCAASLGNYQLIW	TYFCAASTNNYQLIW
TYFCAASGGNYQLIW	TYFCAASQDSNYQLIW
TYFCAASNYQLIW	TYFCAATNTGGLS
TYFCAAEDGNYQLIW	TYFCAARESNYQLIW
TYFCADSNYQLIW	TYFCAASGSNYQLIW
TYFCAASMDWGNYQLIW	TYFCAASARSNYQLIW
TYFCAASGSNYQLIW	TYFCAAKDSNYQLIW
TYFCAASNTGGLS	TYFCAASGNYQLIW
TYFCAASMDSNYQLIW	TYFCAASNYQLIW
TYFCAANPDSNYQLIW	TYFCAASDSNYQLIW
TYFCAASASNYQLIW	TYFCAAMDSNYQLIW
TYFCAAEARNYQLIW	TYFCAAGTGGLS
TYFCAASGYSNYQLIW	TYFCAASGNTGGLS
TYFCAARESNYQLIW	TYFCAASPDSNYQLIW
TYFCAARVDSNYQLIW	TYFCAASSSNYQLIW
TYFCAARANSNYQLIW	TYFCAARNYQLIW
TYFCAANSNYQLIW	TYFCAARAYSNYQLIW
TYFCAAWNTGGLS	TYFCAASEYSNYQLIW
TYFCAARASNYQLIW	TYFCAASRESYQLIW
TYFCAASEDWGNYQLIW	TYFCAASLNTGGLS
TYFCAASEDRGNYQLIW	TYFCAASEGNYQLIW
TYFCAASGNYQLIW	TYFCAASPYSNYQLIW
TYFCAASPDSNYQLIW	TYFCAASGGLS
TYFCAANTGGLS	TYFCAASVGVDSNYQL
TYFCAASDSRSNYQLIW	TYFCAAANTGGLS
TYFCAASAASNYQLIW	TYFCAASEEMDSNYQL
TYFCAPSFQLIW	TYFCAAIGDQLIW
TYFCAASEGNTGGLS	TYFCAASAGNYQLIW
TYFCAAASYSNYQLIW	TYFCAASASSNYQLIW
TYFCAATGGLS	TYFCAASADSNYQLIW
TYFCAASGDNYQLIW	TYFCAASAHSNYQLIW
TYFCAASEETGGLS	TYFCAASAYRNYQLIW
TYFCAASEGSNYQLIW	TYFCAASFVDSNYQLIW
TYFCAADNYQLIW	TYFCAARGGGLS
TYFCAASACGYQLIW	TYFCAARASNYQLIW
TYFCAAMDSNYQLIW	TYFCAASAGSNYQLIW
TYFCAEGSNNYQLIW	TYFCAVDTGGLS
TYFCAASSNYQLIW	TYFCAANSNYQLIW
TYFCAARDGTGGLS	TYFCAARRSNYQLIW
TYFCAASPMNTGGLS	TYFCAASKGSNYQLIW
TYFCAARIGGLS	TYFCAASGTGGLS
TYFCAASLGGNYQLIW	TYFCAAMRGSNYQLIW
TYFCAASVDSNYQLIW	TYFCAASAYSKYQLIW
TYFCAAYSHSNYQLIW	TYFCAASTYSNYQLIW
